# Dust

**Published:** 1893-02

**Authors:** 


### DUST.

It would be well if we could all examine enough specimens of air under the microscope to be able to call up ever afterward vivid pictures of its contents. We would then see the cloud of dust arising from every footfall in a carpet, springing up from every cushioned chair when its occupant leaves it, and flying out from unbrushed clothes and dirty boots. Had we these microscopic eyes to see our real surroundings certain reforms in our households would not be so delayed. There would soon be an end of nailed down carpets that are taken up and beaten but once a year. We would buy very little upholstered furniture, and what we had would be beaten and brushed out of doors much oftener than it now is. We would have no heavy hangings to catch and hold the dust, and, unseen, foul the air at every movement. Our furniture, especially of bedrooms, would be smoother of outline, with fewer dust catchers. We would more intelligently direct our house builders, requiring for one thing perfectly fitting floors, and, for another, fewer mouldings and projections too high to be conveniently cleaned every day, but sure to send down upon us their accumulations at every closing of a door. We should learn that a sweeping is not to be done indoors, but out, the floor covering to be removed for the purpose, and that the removal of dust does not consist in stirring it up only to settle again, but in wiping it up with a slightly dampened cloth, which shall carry the dust with it out of the room. And it is really true, good housewife, that this system, once set running in your house, requires no more labor, take the whole year together, than your present way, and you will be clean all the time instead of once or twice a year.

